# Hypersensitivity to DNA damage in antephase as a safeguard for genome stability

**DOI:** 10.1038/ncomms12618

**Published:** 2016-08-26

**Authors:** Femke M. Feringa, Lenno Krenning, André Koch, Jeroen van den Berg, Bram van den Broek, Kees Jalink, René H. Medema

**Affiliations:** 1 Division of Cell Biology I and Cancer Genomics Center, The Netherlands Cancer Institute, Plesmanlaan 121, Amsterdam 1066 CX, The Netherlands; 2 Hubrecht Institute, The Royal Netherlands Academy of Arts and Sciences (KNAW) and University Medical Center Utrecht, Utrecht 3584CT, The Netherlands

## Abstract

Activation of the DNA-damage response can lead to the induction of an arrest at various stages in the cell cycle. These arrests are reversible in nature, unless the damage is too excessive. Here we find that checkpoint reversibility is lost in cells that are in very late G2, but not yet fully committed to enter mitosis (antephase). We show that antephase cells exit the cell cycle and enter senescence at levels of DNA damage that induce a reversible arrest in early G2. We show that checkpoint reversibility critically depends on the presence of the APC/C inhibitor Emi1, which is degraded just before mitosis. Importantly, ablation of the cell cycle withdrawal mechanism in antephase promotes cell division in the presence of broken chromosomes. Thus, our data uncover a novel, but irreversible, DNA-damage response in antephase that is required to prevent the propagation of DNA damage during cell division.

## Abstract (web summary)

The DNA damage response induces a reversible arrest at different cell cycle stages. Here the authors find that loss of the APC/C inhibitor Emi1 in antephase (late G2) results in hypersensitivity to DNA damage and cell cycle exit at DNA damage levels that induce a reversible arrest in early G2.

## Graphical abstract

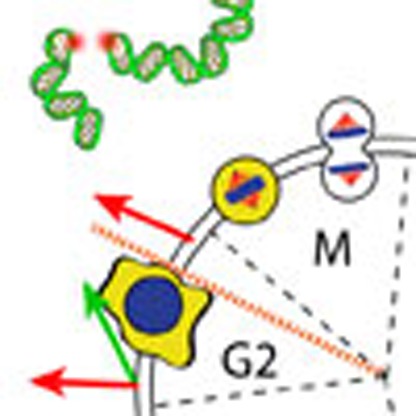

To protect their genome, cells depend on the action of DNA-damage checkpoints that ensure the detection and repair of DNA damage^1^,^2^. These checkpoints can induce a reversible arrest at different stages of the cell cycle to allow for repair to take place before the cell divides^3^,^4^. Functionality of these checkpoints requires accurate coordination between repair, checkpoint signalling and cell cycle progression, such that re-entry into the cell cycle is only allowed once repair has been completed. This is particularly important in G2 phase, since mitotic entry with broken chromosomes poses a direct threat to proper chromosome segregation and genome stability^5^,^6^. In fact, excessive DNA damage in G2 phase can lead to a p53- and p21-dependent exit from the cell cycle, resulting in an irreversible G2 arrest^5^,^7^,^8^,^9^. This way, cell division is prevented if the damage is too severe.

But what happens if a DNA lesion arises after a cell has passed the G2 DNA-damage checkpoint? Several lines of evidence indicate that mitotic cells are refractory to DNA damage, and fail to mount a DNA-damage-induced cell cycle arrest that can prevent cell division^10^,^11^,^12^, and as such damage in mitosis is likely to result in mutated daughter cells.

Contrary to the current view, we show here that the DNA-damage response becomes irreversible already at low levels of DNA damage in late G2. We show that the scheduled loss of early mitotic inhibitor-1 (Emi1) at the end of G2 phase results in hypersensitivity to DNA damage. We find that this novel response to DNA damage is restricted to cells that have separated their centrosomes and display elevated levels of histone H3 Ser10 phosphorylation and Cdk1-dependent phosphorylation. Therefore, we refer to them as cells in antephase. While cells in antephase have been shown to display a reversible arrest in response to various stresses^13^,^14^, we now uncover a novel mechanism that ensures irreversible removal from the cell cycle, when DNA damage occurs at the brink of mitosis. Importantly, this mechanism is crucial to prevent the propagation of damaged chromosomes to G1 daughter cells and to protect genome stability.

## Results

### Cells in antephase show a unique response to DNA damage

**Figure 1:**
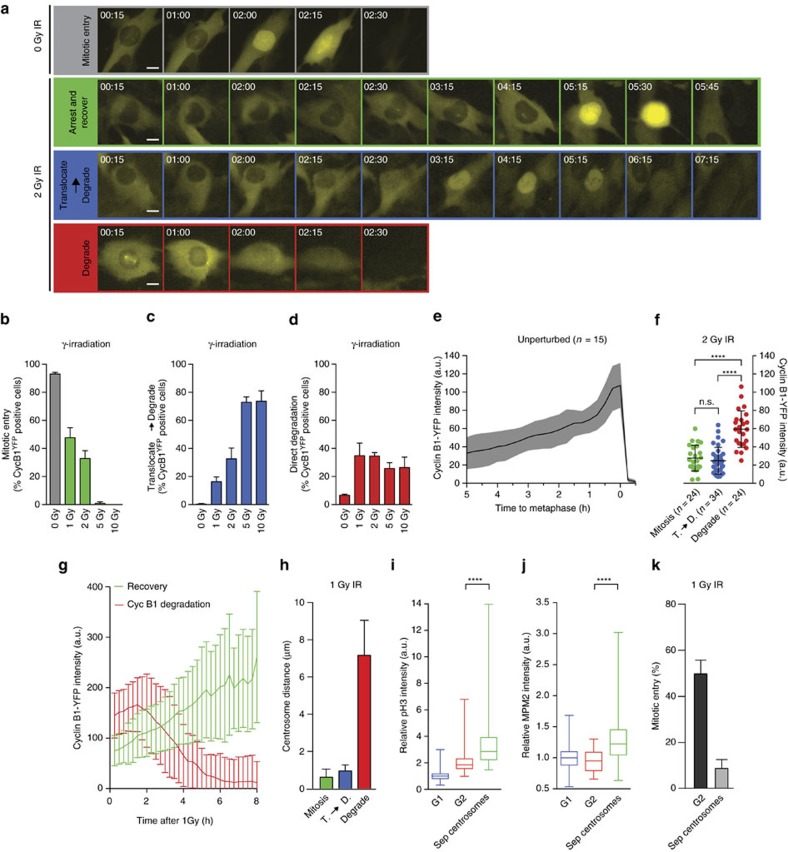
Cells in antephase show a unique response to DNA damage. (**a**) Time-lapse images represent distinct responses of RPE *CCNB*^YFP^-postive cells to ionizing radiation (IR). Time, hh:mm. Scale bars, 10 μm. (**b**–**d**) Quantification of the frequency with which the responses in **a** are observed in Cyclin B1^YFP^-positive cells within 16 h after IR. Mean±s.d. of three independent experiments. (**e**) Cyclin B1^YFP^ intensity during unperturbed G2/M progression in individual cells, and *in silico* aligned at metaphase. Mean±s.d., *n*=15 RPE *CCNB*^YFP^ cells from one experiment. (**f**) Cyclin B1^YFP^ intensity measured 15 min after 2 Gy IR in cells undergoing the indicated responses. Dots represent individual cells (*n*), mean±s.d., results are representative of three independent experiments. ****P<0.0001 (unpaired *t*-test). (**g**) Cyclin B1^YFP^ levels measured in individual cells that either recovered from 1 Gy IR and entered mitosis or that lost Cyclin B1 completely. Mean±s.d. *n*=15 (recovery) and *n*=19 (degradation) RPE *CCNB*^YFP^ cells. (**h**) Centrosome distance measured 15 min after 1 Gy IR in cells undergoing the indicated responses. Mean±s.e.m. of three independent experiments. (**i**,**j**) RPE *CCNB*^YFP^ cells were tracked 5 h by live-cell imaging followed by fixation and staining for MPM2 or pH3. G1, G2 and Cyclin B1-positive cells with separated centrosomes were differentiated based on the live-cell imaging data. Box plots represent *n*>40 cells per condition pooled from three independent experiments. (**k**) Spontaneous recovery after 1 Gy in indicated cell types. Cyclin B1^YFP^-positive cells were separated in two populations based on centrosome status. Mean±s.e.m. of three independent experiments.

To investigate the fate of cells that encountered DNA damage at distinct stages in G2 phase, we performed time-lapse microscopy of untransformed RPE-1 cells with endogenously tagged Cyclin B1^YFP^ (ref. 15). Cyclin B1 expression rises as cells progress through G2 into M, and the absolute level of fluorescence in these cells can be used to derive temporal information, regarding the cell cycle position of the individual cell^16^. Using various doses of ionizing radiation (IR), we find that the subset of Cyclin B1^YFP^-positive cells that recovers from the damage and enters mitosis decreases with increasing dose (Fig. 1a,b). As the dose increases, the recovering fraction is replaced by cells, in which Cyclin B1 translocates to the nucleus (Fig. 1a,c), a process we and others have previously shown to lead to the induction of senescence^7^,^9^,^17^. Interestingly, we find that a subset of Cyclin B1^YFP^-positive cells displays a distinct behaviour. This subset directly degrades Cyclin B1 expression in response to DNA damage (Fig. 1a,d), lacking the prior translocation of Cyclin B1 to the nucleus. The fraction of cells that directly loses Cyclin B1 does not increase with increasing doses of IR (Fig. 1d), in sharp contrast to the dose-dependent nuclear Cyclin B1 retention (Fig. 1c). Moreover, we always observe a small percentage of the undamaged Cyclin B1^YFP^-positive cells that loses Cyclin B1 spontaneously. Remarkably, the cells that directly lose Cyclin B1 have significantly higher levels of Cyclin B1^YFP^ at the moment of irradiation (Fig. 1e–g). In contrast, cells that recover from the damaging event, as well as the cells that translocate Cyclin B1 to the nucleus, express lower levels of Cyclin B1^YFP^ at the moment of irradiation, suggesting that these cells are in the earlier stages of G2 phase (Fig. 1e–g). To further define the cells that directly lose Cyclin B1, we analysed if in this population centrosomes had separated at the moment of irradiation. Strikingly, the vast majority of cells within this population had already started to separate their centrosomes at the time of irradiation, which is normally visible in cells shortly before mitosis (Fig. 1h; Supplementary Fig. 1a). Centrosome separation coincides with a significant increase in levels of phosphorylated H3 (Ser10) and MPM2, both of which are characteristic markers for the onset of mitosis (Fig. 1i,j; Supplementary Fig. 1b). This implies that direct loss of Cyclin B1 upon irradiation is restricted to late G2 or early-prophase cells. We could not observe clear signs of chromosome condensation by 4,6-diamidino-2-phenylindole (DAPI) staining in this population (Supplementary Fig. 1b), most consistent with a cell cycle stage that was previously termed as antephase^13^,^14^.

Subsequently, we tested the consequence of this unique response for the fate of a cell exposed to low-dose irradiation. We used time-lapse imaging to track Cyclin B1^YFP^-positive cells that did or did not already separate their centrosomes at the time of irradiation. We found a clear difference in the fraction of Cyclin B1^YFP^-positive cells without separated centrosomes that managed to recover, when compared with the Cyclin B1^YFP^-positive cells that had already separated their centrosomes (Fig. 1k), indicating the capacity to recover is compromised in antephase. We confirmed that the hypersensitive DNA-damage response in antephase cells is not due to a difference in overall damage or repair signalling, as DNA-damage foci are formed and resolved at similar kinetics in G2 cells that translocate Cyclin B1 to the nucleus, compared with cells that lose Cyclin B1 directly upon irradiation (Supplementary Fig. 1c). Time-lapse imaging of human dermal microvascular endothelium (HMEC-1), mammary gland epithelial (MCF-10a) and human osteosarcoma (U2OS) cells with endogenously tagged Cyclin B1^YFP^ revealed that hypersensitivity to DNA damage in antephase is conserved throughout various cell types (Supplementary Fig. 1d). We therefore conclude that cell fate after DNA damage is regulated in a unique way in antephase cells, which is intrinsically different from the known G2 response. Importantly, this response causes cells in antephase to be highly sensitive to DNA damage.

### DNA damage causes rapid APC/C^Cdh1^ activation in antephase

**Figure 2:**
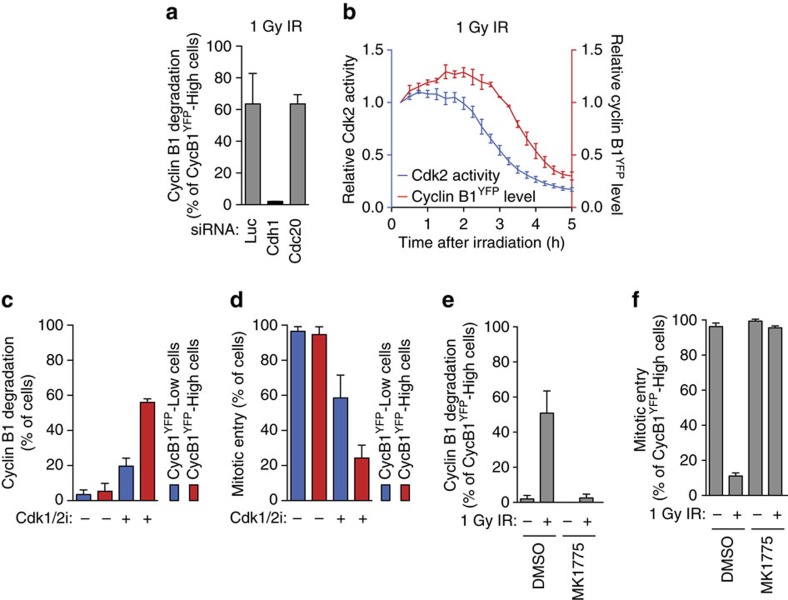
DNA damage causes rapid APC/C^Cdh1^ activation in antephase. (**a**) Direct degradation of Cyclin B1 in antephase cells depleted for luciferase, Cdh1 or Cdc20 that were irradiated with 1 Gy. Antephase cells were selected based on top 25% Cyclin B1^YFP^-expressing cells, measured 15 min after IR. Mean±s.d. of two independent experiments. (**b**) Relative Cdk2 activity and Cyclin B1^YFP^ intensity were measured in individual antephase cells that degraded Cyclin B1 after 1 Gy IR. All time points were normalized to Cdk2 activity and Cyclin B1 level at the first frame, which were set to one. Mean±s.e.m. of three independent experiments. (**c**,**d**) Cyclin B1 degradation (**c**) and mitotic entry (**d**) scored in undamaged G2 and antephase cells within 10 h after wash out of Cdk1 (RO-3306) and Cdk2 (Roscovitine) inhibitors that had been present for 5 h. G2 and antephase cells were separated based on 75% lowest and 25% highest Cyclin B1^YFP^-expressing cells. Mean±s.e.m. of three independent experiments. (**e**,**f**) Cyclin B1 degradation (**e**) and mitotic entry (**f**) scored in antephase cells (selected as in Fig. 2a) within 10 h after IR 1 Gy. Wee1 inhibitor (MK 1775) or dimethylsulfoxide were added immediately after IR. Mean±s.d. of three independent experiments.

Excessive DNA damage results in activation of the Anaphase-promoting complex/Cyclosome together with its co-factor Cdh1 (APC/C^Cdh1^) in G2 phase, to promote the degradation of multiple G2/M targets, including Cyclin B1 (refs 7, 8, 9, 18, 19, 20, 21). This activation of APC/C^Cdh1^ normally occurs several hours after the damage, much later than the onset of Cyclin B1 degradation that we observe in antephase cells. Nevertheless, we set out to test if the direct loss of Cyclin B1 observed after DNA damage in antephase was also caused by APC/C^Cdh1^-dependent degradation. Cells in antephase were selected based on the 25% highest Cyclin B1-expressing cells, which corresponded well with the distinction based on centrosome separation (Supplementary Fig. 2a). Indeed, we could effectively prevent the Cyclin B1 degradation in these cells by depletion of Cdh1 (Fig. 2a). This effect was not seen after depletion of Cdc20, the other co-activator of the APC/C (Fig. 2a; Supplementary Fig. 2b). In addition, we find that the loss of Cyclin B1 is prevented when irradiated antephase cells are treated with the proteasome inhibitor MG-132 (Supplementary Fig. 2c). Immunofluorescent staining of the APC/C targets Aurora A, Cyclin A2 and Plk1 shows that these proteins are also degraded in cells that lost Cyclin B1 (Supplementary Fig. 2d,e). Collectively, these results show that the loss of Cyclin B1 following DNA damage in antephase results from general activation of the APC/C^Cdh1^.

Next, we aimed to find out how APC/C^Cdh1^ can be activated specifically in antephase cells in response to a low-dose irradiation. Activation of APC/C^Cdh1^ in undamaged cells normally occurs in anaphase, following the loss of Cdk activity^22^. Therefore, we investigated whether Cdk inhibition induced by DNA damage precedes the onset of the APC/C^Cdh1^ activation in cells in antephase. Using a previously described live-cell sensor for Cdk2 activity^23^, we measured Cdk2 activity and Cyclin B1 levels in single cells after irradiation (Supplementary Fig. 2f). A clear drop in Cdk2 activity precedes Cyclin B1 degradation in antephase cells irradiated with 1 Gy (Fig. 2b). Next, we tested whether the inhibition of Cdk1 and/or Cdk2 activity by itself would be sufficient to cause APC/C^Cdh1^ activation in late G2. Live-cell imaging of cells in antephase treated with Cdk1 and/or Cdk2 inhibitors revealed that only dual inhibition effectively induced the direct degradation of Cyclin B1, implying that Cdk1 or Cdk2 activity alone is sufficient to keep APC/C^Cdh1^ inactive at the end of G2 phase (Supplementary Fig. 2g,h). More importantly, temporary inhibition of Cdk1 and Cdk2 activity was enough to induce the Cyclin B1 degradation in undamaged cells in antephase, but did not affect early G2 cells in the same way. Instead, G2 cells halted progression to mitosis, but as expected, the majority continued cell cycle progression after they were released from Cdk1/2 inhibition (Fig. 2d; Supplementary Fig. 2i). In contrast, the antephase cells degraded Cyclin B1 and were not able to enter mitosis after wash out of both inhibitors (Fig. 2c,d; Supplementary Fig. 2i). This shows that mere inhibition of Cdk activity in cells that are at the end of G2 phase is sufficient to activate APC/C^Cdh1^. Conversely, inhibition of Wee1, the kinase responsible for inhibitory phosphorylation of Cdk subunits^24^, almost completely prevented the DNA-damage-induced degradation of Cyclin B1 in antephase and promoted mitotic entry (Fig. 2e,f; Supplementary Fig. 2j). Thus, abrupt Cdk inhibition induced by the activation of the DNA-damage checkpoint in antephase causes premature APC/C^Cdh1^ activation, resulting in degradation of Cyclin B1 and cell cycle exit.

### Emi1 acts to maintain recovery competence in G2 cells

**Figure 3:**
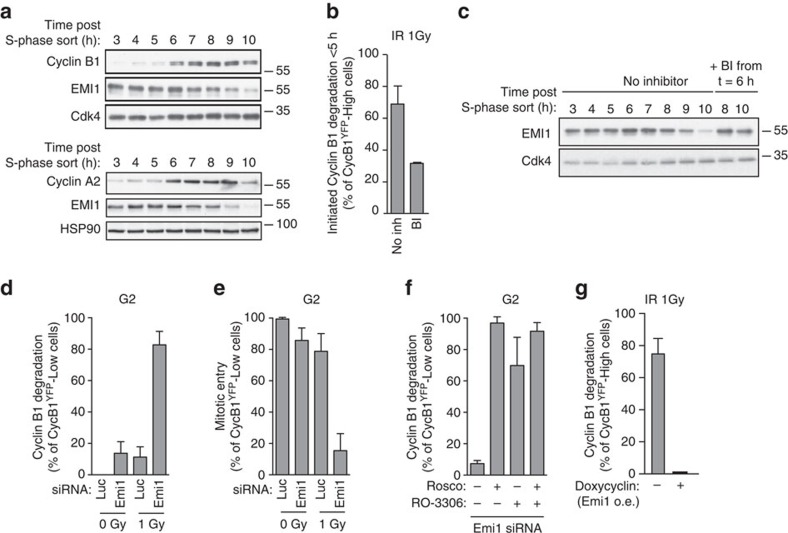
Emi1 acts to maintain recovery competence in G2 cells. (**a**). Western blot showing EMI1, Cyclin A2 and Cyclin B1 protein levels at the indicated time following early-S phase sort. Representative blots of two independent experiments are shown. (**b**) Quantification of antephase cells (selected as in Fig. 2a) that started direct Cyclin B1 degradation within 5 h from IR 1 Gy. BI was added 2,5 h before IR. Mean±s.e.m. of three independent experiments. (**c**) Western blot showing Emi1 protein levels following early-S phase sort in the absence or presence of BI from 6 h after the sort. (**d**,**e**) Cyclin B1 degradation (**d**) and mitotic entry (**e**) within 10 h after 1 Gy IR was analysed in RPE *CCNB*^YFP^ G2 cells (selected as in Fig. 2c) partially depleted for EMI1. Mean±s.d. of three independent experiments. (**f**) Cdk1 (RO-3306), Cdk2 (Roscovitine) or both were inhibited in undamaged RPE *CCNB*^YFP^ cells partially depleted of EMI1. Direct Cyclin B1 degradation of cells in G2 phase at the moment of Cdk inhibition was analysed. Mean±s.d. of three independent experiments. (**g**) EMI1^Turq^ overexpression was induced in RPE *CCNB*^YFP^ cells 2 h before IR 1 Gy using doxycycline. Direct Cyclin B1 degradation was scored in antephase cells (selected as in Fig. 2a). Mean±s.e.m. of three independent experiments.

While our data clearly shows that loss of Cdk activity in antephase causes APC/C^Cdh1^ activation, treatment with Cdk1/2 inhibitors does not activate APC/C^Cdh1^ in all G2 cells. This implies that early G2 cells are protected from a rapid cell cycle exit upon stress-induced Cdk inhibition. A well-known antagonist of APC/C^Cdh1^ activity in S and G2 phase is Emi1 (refs 25, 26). Emi1 is degraded in prophase, prior to nuclear envelope breakdown as a consequence of Plk1- and Cdk1-dependent phosphorylation of Emi1 (refs 27, 28, 29, 30, 31). Excessive DNA damage in G2 cells can cause p21-dependent downregulation of Emi1 resulting in the APC/C^Cdh1^ activation and degradation of its targets^19^,^20^. However, the latter response is limited to cells that contain high levels of damage, and follows only after p21-dependent nuclear retention of Cyclin B1–Cdk complexes^7^,^9^, not matching the fast response we observe in antephase cells. Therefore, we set out to test if Emi1 is needed to protect G2 cells from APC/C^Cdh1^ activation by DNA damage. Using drug-free synchronized RPE-1 Fucci cells (Supplementary Fig. 3a–c), we determined the timing of Emi1 degradation in RPE-1 cells. Staining for Cyclin A2, Cyclin B1 and Emi1 on western blot revealed that Emi1 is indeed degraded before the Cyclins (Fig. 3a). Since, Cyclin A degradation occurs directly after nuclear envelope breakdown^32^,^33^,^34^,^35^, this is most consistent with the degradation of Emi1 shortly before mitosis, similar to previous observations^27^,^28^,^29^,^30^,^31^. Thus, DNA-damage-induced activation of APC/C^Cdh1^ in antephase may be a consequence of limited Cdk activity in cells that have already lost Emi1, and are therefore unable to prevent the APC/C^Cdh1^ activation. To corroborate this notion, we tested whether Plk1-dependent degradation of Emi1 is indeed needed for the unique DNA-damage response we find in antephase cells. Inhibition of Plk1 activity a few hours before irradiation resulted in a clear reduction in direct degradation of Cyclin B1 in antephase cells following DNA damage. This indicates that the scheduled loss of Emi1 at the end of G2 phase is required for the antephase response to DNA damage (Fig. 3b,c). Next, we asked if reduction of Emi1 expression could render the checkpoint irreversible throughout G2. Since, depletion of Emi1 leads to marked phenotypes like rereplication^36^,^37^, we titrated down the concentration of short interfering RNA (siRNA) against Emi1 to establish conditions for depletion of Emi1 that would not perturb cell division in non-damaged cells (Supplementary Fig. 3d,e). Interestingly, partial Emi1 depletion, which hardly affects cell cycle progression in undamaged cells (Supplementary Fig. 3d,e; Supplementary Methods), leads to a very apparent hypersensitivity to DNA damage in G2 (Fig. 3d,e; Supplementary Fig. 3f). The majority of control G2 cells are able to recover from 1 Gy of irradiation, whereas >80% of the Emi1-reduced cells degrade Cyclin B1 at this dose of irradiation, preventing their recovery (Fig. 3d,e; Supplementary Fig. 3f). In addition, reduction of Emi1 in undamaged cells allows the direct Cyclin B1 degradation in all G2 cells, when Cdk activity is chemically inhibited (Fig. 3f; Supplementary Fig. 3g). Conversely, overexpression of Emi1 completely prevents the direct DNA-damage-induced degradation of Cyclin B1 in antephase cells (Fig. 3g). Thus, our data shows that Emi1 acts to sustain checkpoint reversibility in G2, and its degradation at the end of G2 phase results in an irreversible DNA-damage response that ensures a rapid cell cycle exit, even at low levels of DNA damage.

### Hypersensitivity in antephase protects genome stability

**Figure 4:**
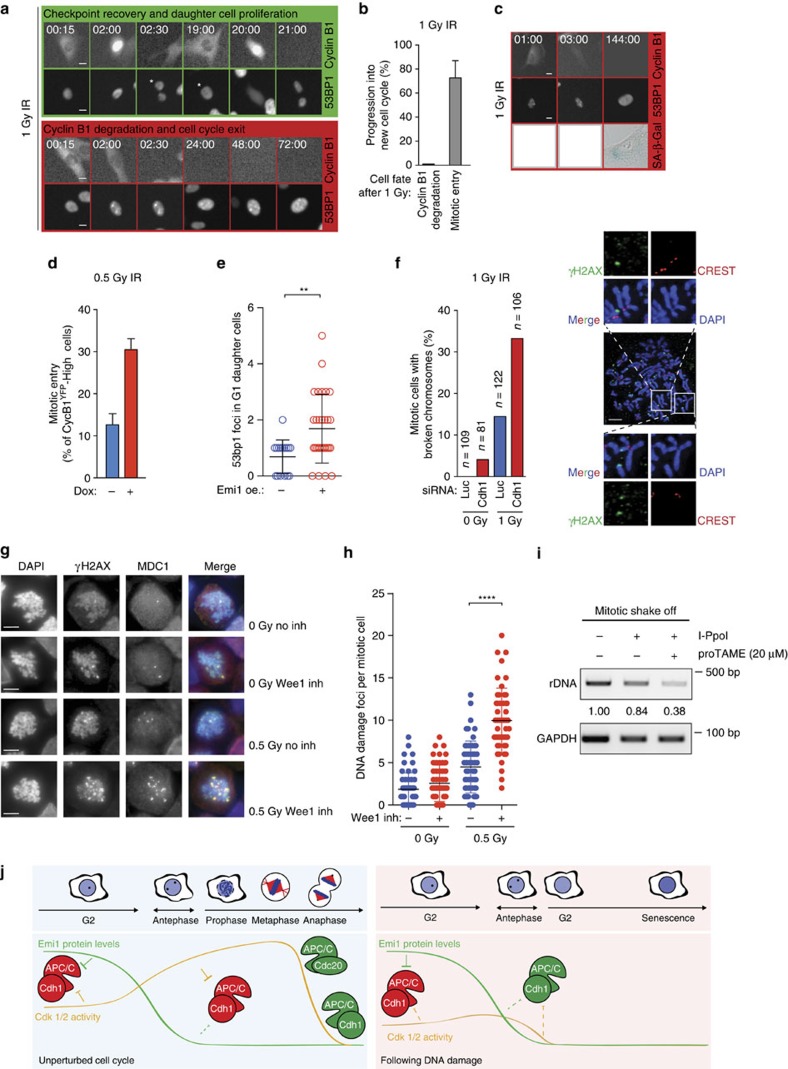
Hypersensitivity in antephase protects genome stability. (**a**) Representative images from time-lapse movies of RPE *CCNB*^YFP^–53BP1^mCherry^ cells following 1 Gy IR. Stills are representative of three independent experiments. Star indicates tracked daughter cell after mitosis. Time, hh:mm. Scale bars, 10 μm. (**b**) Progression into a subsequent cell cycle scored in cells that had degraded Cyclin B1 after 1 Gy and cells that had recovered and entered mitosis. Rebuilding of Cyclin B1 expression within 72 h from IR was used to score cell cycle progression. Mean±s.d. of three independent experiments. (**c**) As in **a**, except cells were followed for 144 h and then stained for SA-β-gal to identify senescent cells. Stills are representative of *n*=16 cells pooled from two experiments. Time, hh:mm. Scale bars, 10 μm. (**d**) Mitotic entry of antephase cells (selected as in Fig. 2a) in presence or absence of EMI1^Turq^ overexpression. Mean±s.e.m. of three independent experiments. (**e**) Quantification of 53BP1^mCherry^ foci in G1 daughter cells 3 h after mitosis in the presence or absence of Emi1 overexpression, induced as in **d**. *n*=16 (-Emi1 oe) and *n*=29 (+Emi1 oe) number of cells analysed, pooled from three independent experiments. **P<0.01 (unpaired *t*-test) (**f**). Quantification of mitotic cells with broken chromosomes in Luc- or Cdh1-depleted cells that entered mitosis within 4 h after IR. Average of *n*, number of cells analysed in two independent experiments. Representative images show broken (bottom) or intact (top) chromosomes. Scale bar, 5 μm. (**g**) Representative stills from mitotic cells following mock or 0.5 Gy IR stained for yH2AX and MDC1 to quantify double-positive DNA damage foci. Scale bars, 10 μm. (**h**) Number of double-positive foci per mitotic cell (as in **g**). Wee1 inhibitor was added just after mock IR or 0.5 Gy. *n*>100 per sample pooled from three independent experiments. ****P<0.0001 (unpaired *t*-test). (**i**) PCR on genomic DNA from mitotic cells at the I-PpoI break site in the 45 S locus (rDNA) or GAPDH. Numbers indicate the quantification of rDNA band intensity normalized for DNA loading using the GAPDH control. (**j**) Model to show why cells in antephase are hypersensitive to DNA damage and how damaged antephase cells exit the cell cycle.

Our observation that cells in antephase directly degrade Cyclin B1 after low doses of irradiation indicates that antephase cells withdraw from the cell cycle in the presence of low levels of DNA damage. Indeed, none of the cells in antephase that degraded Cyclin B1 after exposure to 1 Gy were able to proliferate within 72 h after damage (Fig. 4a,b). In contrast, 75% of the G2 cells that recovered from this dose also progressed into subsequent cell divisions within 72 h (Fig. 4a,b). Moreover, time-lapse analysis of individual cells that were followed for 5 days and subsequently stained for senescence-associated β-galactosidase (SA-β-gal) activity, confirmed that antephase cells enter a senescent state in response to a low-dose irradiation (Fig. 4c).

While high doses of irradiation lead to extensive checkpoint activation, including long-lasting Cdk inhibition, and high levels of p53 and p21, low doses of irradiation induce a much milder checkpoint response only temporarily inhibiting Cdk activity^7^. We therefore hypothesized that rapid cell cycle exit of cells damaged in antephase would be especially important after low levels of damage, since these cells will likely progress into mitosis as soon as Cdk activity is restored. In such cases, the little time available for repair could pose a serious threat to genomic integrity, and this could be compensated by a rapid cell cycle exit to prevent cell division with broken chromosomes. Since, we could prevent the DNA-damage-induced Cyclin B1 degradation in antephase cells by overexpression of Emi1, we asked if this could promote mitotic entry. Indeed, we find that mitotic entry is restored in antephase cells expressing Emi1 (Fig. 4d; Supplementary Fig. 4a). Importantly, the increased mitotic entry of antephase cells is associated with increased reappearance of 53BP1 foci in G1 daughter cells (Fig. 4e), indicating that loss of the antephase-specific response to low levels of DNA damage results in carryover of damaged DNA to the daughter cells. Similarly, the number of mitotic cells with broken chromosomes observed in Cdh1-depleted cells after 1 Gy irradiation was twice that seen in control cells (Fig. 4f). This difference is not a consequence of altered damage signalling caused by Cdh1 depletion, since the number of breaks was similar in luciferase- and Cdh1-depleted cells that were pushed into mitosis by the addition of caffeine (Supplementary Fig. 4c). To further test the robustness of the hypersensitive response in antephase, we collected cells that entered mitosis following 0.5 Gy or mock irradiation, and quantified foci positive for phosphorylated H2AX (γH2AX) and MDC1. Cells that entered mitosis following 0.5 Gy of irradiation displayed only a slight increase in DNA-damage-associated foci compared with undamaged cells. In contrast, cells that were irradiated and co-treated with a Wee1 inhibitor to prevent Cdk inhibition entered mitosis with a considerable higher number of DNA-damage-associated foci (Fig. 4g,h). Together, this shows that the hypersensitive DNA-damage response in antephase accurately prevents the mitotic progression of damaged cells. Finally, we used the I-PpoI nuclease to track DNA damage at specific loci. This nuclease cuts in the ribosomal DNA (rDNA) in addition to several other locations in the genome^38^. Using PCR primers flanking the rDNA break sites, we observed only a slight reduction in intact rDNA in mitotic cells that recovered spontaneously from the induced damage. In contrast, when the antephase response was acutely abrogated using the APC/C inhibitor proTAME, a marked loss of intact rDNA was detected in cells that entered mitosis, indicating that many of the cells entered mitosis with residual breaks (Fig. 4i; Supplementary Fig. 4b,d). In conclusion, this previously unidentified cell cycle exit mechanism in antephase is important to prevent cell division with broken chromosomes.

## Discussion

The fate of a cell after DNA damage is a result of the complex interplay between DNA repair, checkpoint signalling and cell cycle progression. It is still largely unknown how cell fate after DNA damage is determined. Our data show that cells in antephase have a very unique, irreversible response to DNA double strand breaks that can be engaged by minimal amounts of damage. The definite irreversibility of the DNA-damage response in antephase cells is in sharp contrast to the reversible cell cycle arrests that act in other stages of the cell cycle^3^. This response has important consequences for the fate of damaged cells in antephase, since low levels of damage already lead to an irreversible cell cycle exit.

Both terms early prophase and antephase have been used to refer to cells at the G2/M transition^13^,^39^,^40^. The term antephase was defined as stage in late G2, before visible signs of DNA condensation. While we do see Ser10-phosphorylated histone H3 appear in these cells, we do not see any visible signs of DNA condensation. We show that the cells we refer to as being in antephase have started centrosome separation and stain positive for mitotic phosphorylation sites, indicating that they are well on their way to mitosis. It should be noted that the current definition of antephase only clarifies when antephase ends, namely at the start of visible DNA condensation. It does not define when antephase starts. Our data indicate that the start of centrosome separation and/or degradation of Emi1 could serve as good markers to define the onset of antephase.

Interestingly, early-prophase or -antephase cells were previously reported to revert back into an interphase-like state upon various stresses, but assumed to re-enter the cell cycle afterwards^41^,^42^,^43^. For instance, mitotic entry is delayed when cells were treated with microtubule poisons^44^. This fully reversible arrest was shown to be dependent on checkpoint with FHA and RING finger domains (CHFR) and p38 signalling, and defined as the antephase checkpoint^40^. The antephase response we describe here is fundamentally different from this previously described antephase checkpoint. We find that DNA double strand breaks induce an irreversible response when they occur in antephase. Importantly, we show that cells in antephase are hypersensitive to DNA damage, when compared with cells in earlier stages of G2 phase. Moreover, our results reveal the underlying mechanism, and emphasize the importance of this response in protecting genomic integrity. We find that reinstalling reversibility of the DNA-damage response in antephase cells results in an increased carryover of DNA double strand breaks from mother to daughter cells.

In addition, we demonstrate an essential role for Emi1 in the DNA-damage checkpoint in G2 phase in that it acts to maintain checkpoint reversibility, thereby reducing sensitivity of a cell to DNA damage by allowing time for DNA repair and subsequent checkpoint recovery. As such, Emi1 could be particularly important for post-replication repair, needed to repair lesions that are created during replication in S phase^45^,^46^. Thus, removal of cells that encounter DNA damage shortly before mitosis from the cell cycle is an important function of APC/C^Cdh1^, and impairing this function could promote genomic instability. In this respect, it is of interest to note that both loss of Cdh1 and overexpression of Emi1 have been reported in several tumour types^21^,^26^,^47^,^48^,^49^.

## Methods

### Time-lapse microscopy and irradiation

Cells were grown in Lab-Tek II chambered coverglass (Thermo Scientific) in Dulbecco's Modified Eagle Medium/Nutrient Mixture F-12 (DMEM/F12), which was replaced by Leibovitz's L-15 (Gibco) CO_2_-independent medium just before imaging. Images were obtained using a DeltaVision Elite (applied precision) maintained at 37 °C equipped with a 10 × 0.4 numerical aperture (NA) or 20 × 0.75 NA or 40 × 1.35 NA lens (Olympus) and cooled CoolSnap CCD camera. Only for time-lapse imaging of the RPE Fucci cells, cells were grown in 96-wells plate in DMEM/F12 during filming. Images were obtained using a CCD microscope (Zeiss AxioObserver.Z1 gemot.) maintained at 37 °C and 5% CO2 equipped with a 10 × /0.25 Achroplan Ph1 lens and cooled Hamamatsu ORCA R2 Black and White CCD-camera. Image analysis was done using ImageJ software. Cells were γ-irradiated using a Gammacell Exactor (Best Theratronics) with a ^137^Cs source.

### ImageJ macros for quantification of DNA-damage foci

Monitoring DNA-damage foci requires following individual cells over time. However, faithful automatic tracking of the highly motile RPE cells in densely covered samples proved to be unfeasible. Therefore, a hybrid approach was taken, where single cells were first manually isolated using an in-house developed cell tracking macro in ImageJ (NIH), after which the DNA damage response was fully automatically quantified with a second ImageJ macro. User-assisted tracking and segmentation of single cell (nuclei) is facilitated as follows: *Z* stacks are converted to two dimensional using a maximum intensity projection. For every frame, a square region of defined size around the *x*,*y* position of the mouse cursor is copied from the original three-dimensional/four-dimensional image stack into a new image stack. The size of the cropped square has to be chosen large enough to fully encompass the cell (nucleus) of interest, which is now centred in the newly generated movie. When holding down the mouse button the time series advances at a desired speed, allowing accurate manual tracking.

Single cell (nuclei) are isolated from such tracked-cell movies in the following manner: three-dimensional time-lapse movies are projected to two dimensional via one of several user-defined methods: maximum intensity projection, automatically select sharpest slice, manually select a slice or via a ‘extended depth of field' algorithm.

Region of interests (ROIs) of candidate nuclei are automatically obtained throughout the image stack by auto-thresholding an outlier-removed median-filtered (0.7 μm radius) *z* projection of the nuclei channel, followed by a watershed command to separate touching nuclei and particle analyser run with size (>4 and <40 μm^2^, and circularity (>0.25) constraints. In each frame, the distances of all detected ROIs to the *x*,*y* center of the image are calculated, after which all except the closest ROI are removed. This procedure thus yields a movie with a single ROI per frame, tightly surrounding the nucleus followed with the mouse in the manual tracking macro.

In the detection of DNA-damage foci, the foci threshold level is defined by the signal-to-noise ratio (SNR): a (user-set) factor times the s.d. of the background fluorescence intensity of the nucleus. The latter property is approximated by first crudely removing signal outliers (the foci), and then taking the median and s.d. of the lower ∼80% pixel values in the ROI, respectively. The background intensity is subtracted using a Difference of Gaussians filter. Foci are then identified as regions of adjacent pixels with grey values, exceeding the SNR threshold and area larger than a certain minimum. In the procedure, the SNR is the only user-defined parameter, and is iteratively optimized by comparing the detected foci with the original signal in an overlay image.

The evolution of the DNA-damage foci is quantified by reporting the number of foci, foci intensity, foci area, and the total signal above threshold for each time frame.

### FACS-sort and SA-β-gal

Cells were trypsinized and resuspended in Leibovitz's L-15 medium for sorting, using a Becton Dickinson FacsAria Sorter or a Beckman Coulter Moflo Astrios. G2 cells were sorted based on Cyclin B1-YFP signal and replated for filming. RPE Fucci S phase cells were sorted based on Azami-Green and Kusabira-Orange double-positive signal, and replated for filming, fluorescence-activated cell sorting (FACS) and western blot samples at indicated time points after the sort. For FACS analysis of Propidium Iodide (PI) profiles after the double-positive Fucci sort, cells were fixed in ice-cold ethanol at indicated time points after the sort. Cells were washed with 1 × PBS before they were resuspended in 1 × PBS+RNAse and PI (10 mM, Sigma). PI profiles were analysed using a Becton Dickinson FACSCalibur analyser. To detect SA-β-gal activity cells were fixed for 5 min using 2% formaldehyde+0,2% gluteraldehyde in PBS within the Lab-Tek II chambered coverglass (Thermo Scientific) after 6 days of live-cell imaging. Cells were washed three times with × PBS before overnight (16 h) incubation in staining solution (X-gal in dimethylformamide (1 mg ml^−1^), citric acid/sodium phosphate buffer at pH6 (40 mM), potassium ferrocyanide (5 mM), potassium ferricyanide (5 mM), sodium chloride (150 mM) and magnesium chloride (2 mM)) at 37 C (not in a CO2 incubator). Cells are washed with 1 × PBS and colour images to detect blue staining were taken using a CCD microscope equipped with a Zeiss AxioCam colour camera (Axiocam HRc).

### Immunodetection and chemicals

For immune fluorescent staining, cells were fixed with 3% formaldehyde for 5 min and permeabilized with 0,2% TritonX for 5 min before blocking in 3% fetal bovine serum (BSA) in 1 × PBS supplemented with 0,1% Tween (PBST) for 1 h. Cells were incubated overnight at 4 °C with primary antibody in PBST with 3% BSA, washed three times with PBST, and incubated with secondary antibody and DAPI in PBST with 3% BSA for 2 h at room temperature (RT). Immunofluorescent staining of Cyclin A, Aurora A and Plk1 was performed after formaldehyde fixation of cells that were tracked by live-cell imaging within the Lab-Tek II chambered coverglass (Thermo Scientific). Immunofluorescent staining of γH2AX and MDC1 DNA-damage foci was performed after formaldehyde fixation of mitotic cells that were collected 2 h after IR by shake-off. Nocodazole was added from 1 h after IR to entrap cells in mitosis and Wee1 inhibitor was added directly after IR where indicated.

For western blot analysis, equal amounts of proteins were separated by SDS–polyacrylamide gel electrophoresis electrophoresis followed by semi-dry transfer to a nitrocellulose membrane. Membranes were blocked in 5% milk in PBST for 1 h at RT before overnight incubation with primary antibody in PBST with 3% BSA at 4 °C. Membranes were washed three times with PBST followed by incubation with secondary antibody in PBST with 5% milk for 2 h at RT. Antibodies were visualized using enhanced chemiluminescence (ECL) (GE Healthcare). Uncropped western blot scans can be found in Supplementary Fig. 5.

The following primary antibodies were used in this study: anti-phospho-H3 (06-570 Upstate, 1/500), anti-γH2AX (ser139p; 05–636 Upstate, 1/500), anti-MPM2 (05–368 Ubi, 1/500), anti-MDC1 (ab11171 Abcam, 1/500), anti-Emi1 (376600 Novex, 1/500), anti-Cdh1 (DH01; ms 1116-p1 Neo, 1/500), anti-Cyclin B1 (GNS1; sc-245 Santa Cruz, 1/500), anti-Cdk4 (C-22; sc-260 Santa Cruz, 1/1,000), anti-HSP90 (sc7947 Santa Cruz, 1/1,000), anti-UBF (F-9; sc-13125 Santa Cruz, 1/500), anti-Cyclin A2 (H432; sc 751 Tebu, 1/1,000), anti-tubulin gamma (GTU-88; ab11316 Abcam, 1/1,000), anti-CREST serum (CS1058 Cortex Biochem, 1/1,000), anti-IAK1 (Aur A; 3092 Cell Signaling, 1/500), anti-GFP (homemade, gift from Geert Kops, 1/1,000), anti-γH2AX (2577 Cell Signaling, 1/500) and anti-BrdU (ab6326–250 Abcam, 1/500). The following secondary antibodies were used for western blot experiments: peroxidase-conjugated goat anti-rabbit (P448 DAKO, 1/1,000), goat anti-mouse (P0447 DAKO, 1/1,000) and rabbit anti-goat (P160 DAKO, 1/1,000). Secondary antibodies used for immunofluorescence and FACS analysis were goat anti-rabbit/Alexa 488 (A_11008 Molecular probes, 1/1,000), goat anti-mouse/Alexa 568 (A11004 Molecular probes, 1/1,000) and goat anti-rat/Alexa 647 (A21247 Molecular probes, 1/600).

Chemicals used in this study: RO-3306 (used at 5 μM) and Roscovotine (used at 25 μM; Calbiochem). Nocodazole (used at 250 μM), caffeine (used at 5 mM), doxocycline (used at 1 mM), Wee1 inhibitor MK-1775 (used at 3 μM), MG-132 (used at 5 μM) and Aphidicolin (used at 0.2 or 0.4 μM) were purchased at Sigma.

### Cell lines

hTert-immortalized retinal pigment epithelium (RPE-1) cells (ATCC) were maintained in DMEM/F12 (Gibco) supplemented with ultraglutamine, antibiotics and 10% fetal calf serum. RPE-1 cells in which a fluorescent tag was introduced in one allele of Cyclin B1 (RPE *CCNB*^YFP^) have been described before^15^. HMEC-1 cells and MCF10a cells were maintained in DMEM/F12 (Gibco) supplemented with ultraglutamine, antibiotics, EGF (20 ng ml^−1^), hydrocortisone (500 ng ml^−1^) and insulin (10 μg ml^−1^). U2OS cells were maintained in DMEM (Gibco) supplemented with ultraglutamine, antibiotics and 6% fetal calf serum. A fluorescent tag was introduced in one allele of Cyclin B1 (*CCNB*^YFP^) in HMEC-1, MCF10a and U2OS cells AAV virus expressing a targeting sequence with 959 bp homology upstream-eYFP- and 1,256 bp homology downstream in the *CCNB* gene was collected 2 days after transfection of HEK293 cells with pAAV-eYFP together with pRC and pHelper plasmids. Indicated cell types were infected with the targeting AAV virus and eYFP-positive cells were FACS sorted and plated to collect single-cell clones. Clones were selected based on correct eYFP expression on the centrosomes and degradation at the end of mitosis.

To make RPE1-Fucci cells HEK293 cells were transfected with Fucci constructs, which have been described before^50^ using X-tremeGENE (Roche) according to manufacturer's protocol. RPE-1 cells expressing ecotropic receptor, described before^51^, were infected after 2 days for 24 h and double-positive Fucci cells were sorted by FACS two weeks later.

The venus tag in the previously described CSII-EF-DHB-venus contruct^23^ was exchanged for mCherry to generate RPE *CCNB1*^YFP^ DHB mCherry-expressing cells. HEK293 cells were transfected with CSII-EF-DHB-mCherry using X-tremeGENE (Roche) according to manufacturer's protocol. RPE *CCNB1*^YFP^ cells were infected after 2 days for 24 h and mCherry-positive cells were sorted out by FACS 2 weeks later. RPE *CCNB1*^YFP^-Turq-Emi1 cells were generated as described for the DHB-mCherry-expressing cells above, except that individual grown clones were selected to generate a monoclonal cell line. The venus tag in the previously described pT7-Venus-Emi1 construct^29^ (purchased from addgene, #39854) was exchanged for mTurquoise. Subsequently, Cas9 was exchanged for mTurq-Emi1 in the all in one dox-inducible lentiviral pCW-Cas9 construct (purchased from addgene, #50661), using Nhe1 and BamH1 restriction sites. RPE *CCNB1*^YFP^-53BP1-mCherry and RPE *CCNB1*^YFP^- Turq-Emi1- 53BP1-mCherry cells were generated using the 53BP1-mCherry construct described before^52^. Amphotropic Phoenix cells were transfected with 53BP1-mCherry using X-tremeGENE (Roche) and virus was used to generate 53BP1 mCherry-positive cells as described above. Positive cells were sorted out by FACS 2 weeks later. All cell lines described above were tested negative for mycoplasma contamination.

### siRNA information

ON-TARGETplus SMARTpool siRNAs targeting luciferase (GL2 duplex), Cdh1/FZR1, Cdc20 and EMI1/FBXO5 were from Thermo Scientific, and were transfected using RNAiMAX (Life Technologies) according to the manufacturer's protocol. All transfections were performed 24 h before experiments.

### Sample sizes

For all experiments where phenotypic outcome was quantified at least 50 cells per condition in each independent biological replicate were scored, *n*≥50. Exceptions are Fig. 1h. *n*=6–27—Supplementary Fig. 1c. *n*=31–52 (RPE1), *n*=18–41 (U2OS), *n*=5–40 (MCF10a) and *n*=21–45 (HMEC)—Fig. 2a. *n*=34–52—Fig. 2b. *n*=6–15—Supplementary Fig. 2c. *n*>38—Fig. 3b. *n*=22–59—Fig. 4b. *n*=18–22—Fig. 4d. *n*=19–49—Supplementary Fig. 4b *n*>32.

### Chromosome spreads

Luciferase or Cdh1-depleted RPE-1 cells were mock irradiated or irradiated with 1 Gy followed by nocodazole addition 1 h after IR to retain cells in mitosis, but exclude cells that were damaged in mitosis. Caffeine was added to control samples to push G2 cells into mitosis with double strand breaks (DSBs) as a positive control. Mitotic RPE-1 cells were collected by shake-off 4 h after IR, washed in 1 × PBS and treated for 25 min with 75 mM KCl at 37 °C. Cells were spun on coverslips at 1,800 r.p.m. for 5 min in a Cytospin 4 (Thermo Scientific). Cells were permeabilized for 1 min with PEM buffer (100 mM PIPES, 2 mM EGTA, 1 mM MgSO_4_; pH 6.8) containing 0.25% Triton X-100 and then fixed for 10 min in 4% paraformaldehyde containing 0.1% Triton X-100. Fixed cells were washed three times in 1xPBS containing 0.1% Tween-20 and then blocked for 30 min in PEM/3% BSA/0.1% Tween-20. Antibody incubations (ACA and γH2AX) were performed overnight at 4 °C. Cells were washed three times in 1 × PBS containing 0.1% Tween-20, and then incubated with secondary antibodies and 0.1 μg ml^−1^ DAPI in PEM/3% BSA/0.1% Tween-20 for 2 h at RT. After three washing steps the coverslips were mounted on microscopic slides with Prolong Gold (Invitrogen) and stored at 4 °C. DNA breaks were quantified based on DAPI and γH2AX signal in chromosome spreads.

### I-PpoI and PCR

RPE-1 hTERT with doxycycline-inducible HA-FKBP(DD)-I-PpoI were synchronized in late G2, using a double thymidine block followed 8 h release in the presence of doxycycline (1 μg ml^−1^). One hour before the addition of nocodazole (250 ng ml^−1^) to trap cells in mitosis, shield-1 (0.5 μM) was added to stabilize the I-PpoI endonuclease. Four hours after the addition of nocodazole in the absence and presence of ProTame (20 μM), cells were collected by a mitotic shake-off. Genomic DNA was extracted using DirectPCR-Cell (Viagen Biotech) according to the manufacturers protocol. PCR products were size-fractionated by gel electrophoresis and visualized by ethidium bromide staining. Primers used in this study:

rDNA (I-PpoI) forward: 5′-GCCTAGCAGCCGACTTAGAA-3′/reverse: 5′-CTCACCGGGTCAGTGAAAAA-3′

GAPDH forward: 5′-TCGGTTCTTGCCTCTTGTC-3′/reverse: 5′-CTTCCATTCTGTCTTCCACTC-3′

### Data availability

The authors declare that all data supporting the findings of this study are available within the article and its Supplementary Information Files.

## Additional information

**How to cite this article:** Feringa, F. M. *et al.* Hypersensitivity to DNA damage in antephase as a safeguard for genome stability. *Nat. Commun.* 7:12618 doi: 10.1038/ncomms12618 (2016).

## Supplementary Material

Supplementary Information Supplementary Figures 1-5 and Supplementary Methods

Peer review file
