# Supplementary material for: Hypersensitivity to DNA damage in antephase as a safeguard for genome stability
Source: Nat Commun. 2016 Aug 26;7:12618. doi: 10.1038/ncomms12618 (PMC5007458; doi:10.1038/ncomms12618)
Supplement: Supplementary Information — Supplementary Figures 1-5 and Supplementary Methods [file ncomms12618-s1.pdf]

Figure S1

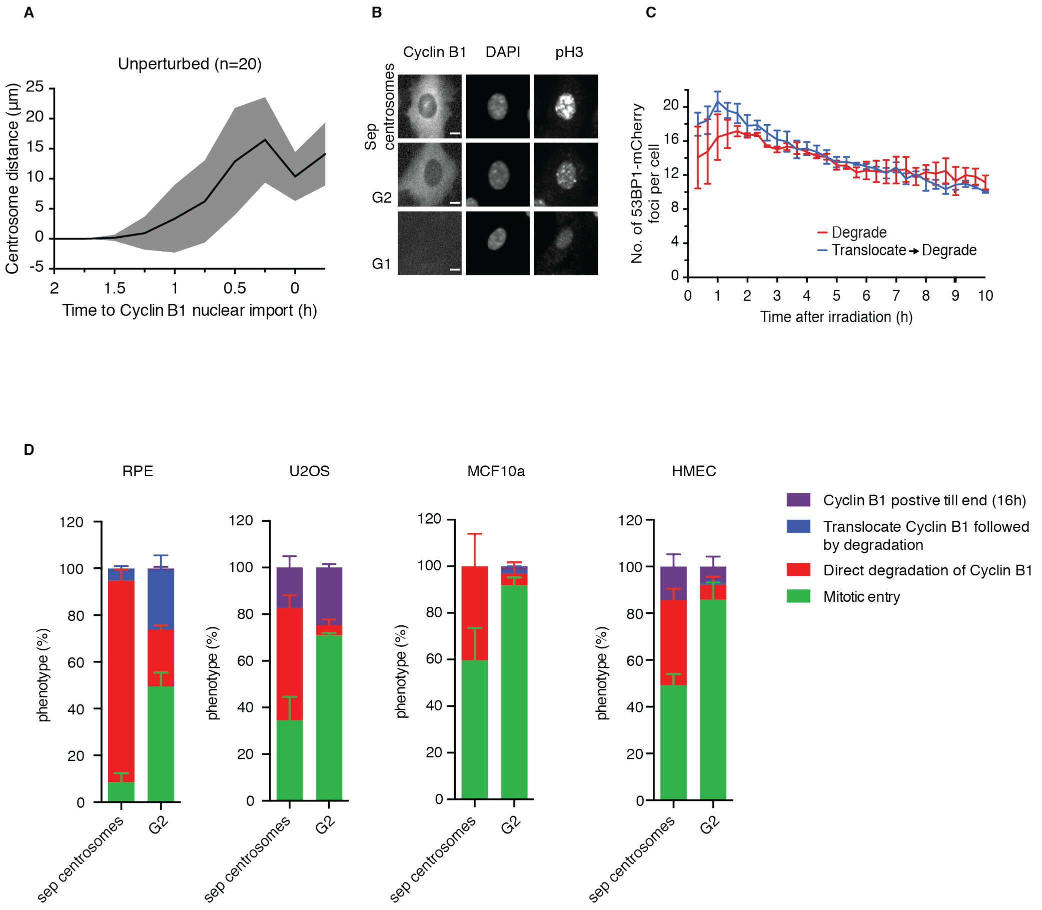

Supplementary figure 1: Cells in antephase show a unique response to DNA damage a. Centrosome separation measured in unperturbed RPE *CCNB*^{YFP} cells, and in silico aligned to nuclear import of Cyclin B1. Mean ± s.d. n=20 RPE *CCNB*^{YFP} cells from one experiment. **b.** Stills represent RPE *CCNB*^{YFP} cells identified by live cell imaging followed by fixation and staining for pH3 ser10 and DAPI to quantify pH3 signal in G1, G2 and Cyclin B1-positive cells with separated centrosomes. Quantification in Figure 1i,j. Scale bars, 10µm. **c.** DNA damage foci were tracked in time-lapse movies of individual RPE *CCNB*^{YFP} cells with 53BP1^{mCherry} expression using automated foci analysis (see methods). Number of foci per cell at indicated time-points after 2 Gy IR is shown. Mean ± s.e.m. of three independent experiments. **c.** Quantification of indicated phenotypes following 1 Gy in RPE, U2OS, MCF10a and HMEC cells expressing endogenously tagged Cyclin B1 with or without separated centrosomes. Mean ± s.e.m. of three independent experiments.

Figure S2

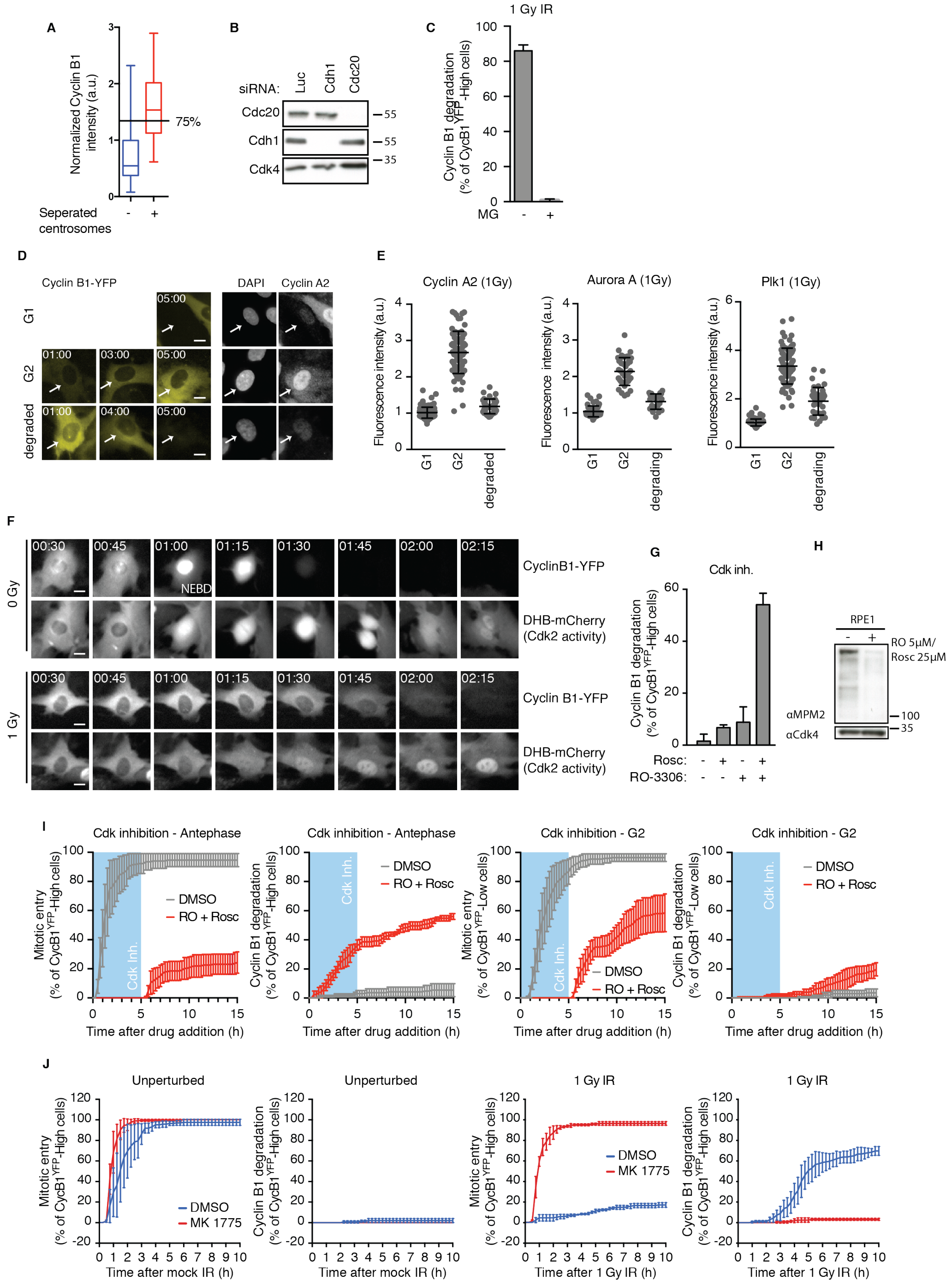

Supplementary figure 2: DNA damage causes rapid APC/C^{Cdh1} activation in antephase a. Quantification of Cyclin B1^{YFP} intensity in cells with or without separated centrosomes. Only Cyclin B1^{YFP}-positive cells were included and the line indicates the border between 75% lowest Cyclin B1 expressing cells and 25% highest Cyclin B1 expressing cells. n>100 cells pooled from two independent experiments. **b.** Cdh1 and Cdc20 knockdown in RPE *CCNB*^{YFP} cells. **c.** Quantification of Cyclin B1 degradation in antephase cells (selected as in fig. 2a) after 1 Gy IR in the presence or

absence of proteasome inhibitor MG132. Mean ± s.e.m. of three independent experiments. **d.** Representative stills of Cyclin B1 expression in a G1 and G2 cell or in an antephase cell that degrades Cyclin B1 following 1Gy. Cells were fixed after 5h and stained for Cyclin A2, Aurora A and Plk1. Scale bars, 10µm. **e.** Quantification of fluorescence intensity in G1, G2 and antephase cells that degraded Cyclin B1 selected based on 5h live cell imaging (Fig 2d). n=22-82 cells per condition, pooled from two (Plk1) or three (Cyc A and Aur A) independent experiments. Error bars, mean ± s.d. **f.** Time-lapse images of RPE *CCNB*^{YFP} DHB^{mCherry} cells show the higher cytoplasmic signal before mitosis and the increased nuclear signal after mitosis in unperturbed growing cells. Relative Cdk2 activity is determined by dividing the mean Cytoplasmic intensity over the mean nuclear intensity in every frame. Scale bars, 10µm. **g.** Quantification of Cyclin B1 degradation in antephase cells (selected as in fig. 2a) after addition of indicated inhibitors. Mean ± s.d. of three independent experiments. **h.** Western blot of asynchronous RPE cells following 1h treatment with RO-3306 5µM and Roscovotin 25µM. **i.** Line graphs of the data presented in figure 2c and d showing cumulative mitotic entry or cumulative Cyclin B1 degradation. Mean ± s.d. of three independent experiments. **j.** Line graphs of the data presented in figure 2 e and f showing cumulative Cyclin B1 degradation. In addition cumulative mitotic entry is shown. Mean ± s.d. of three independent experiments.

Figure S3

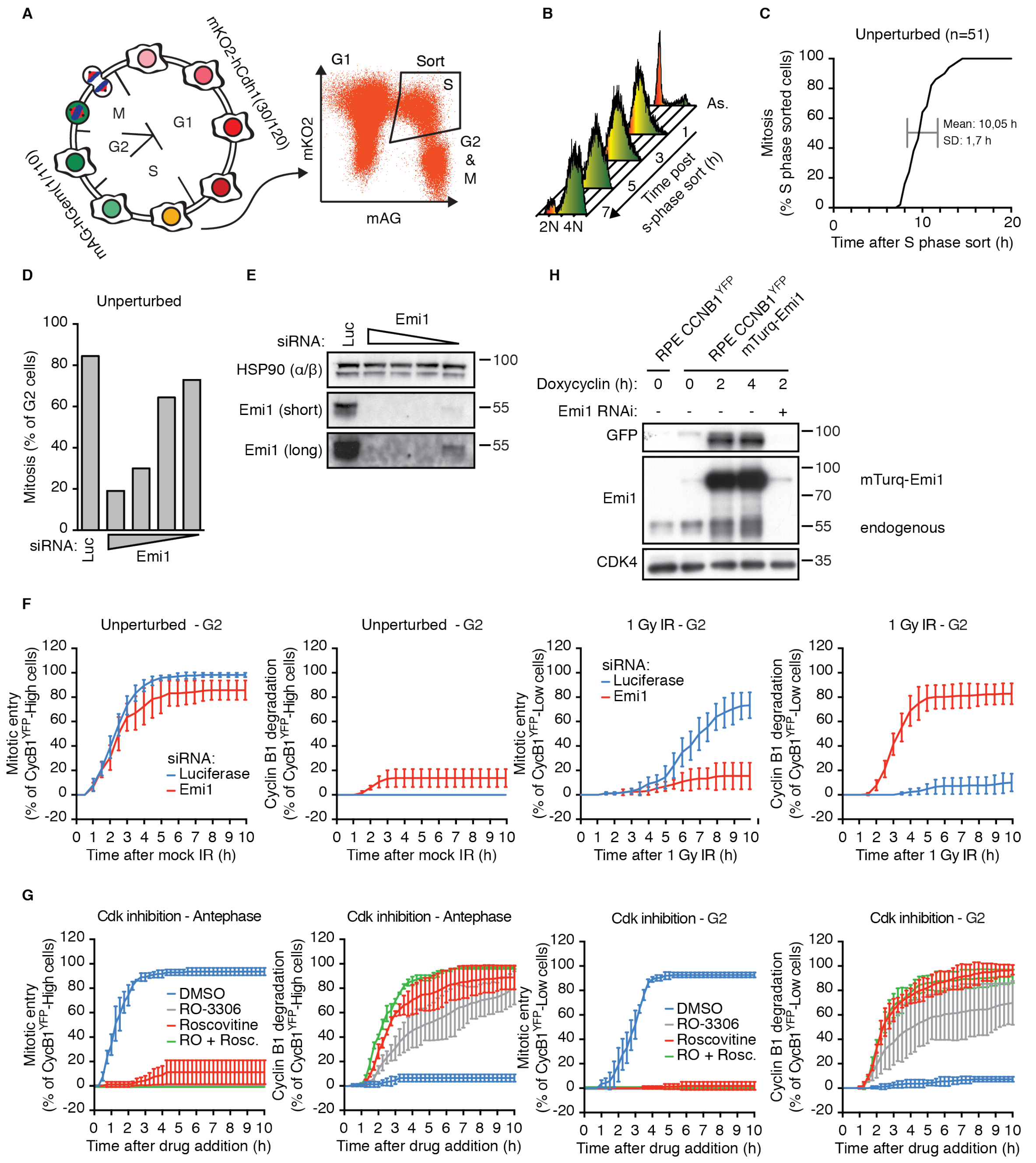

Supplementary figure 3: Emi1 acts to maintain recovery competence in G2 cells

a. Scheme representing how early S phase cells were obtained by FACS sorting of double positive RPE Fucci cells (Azami-Green (AG) and Kusabira-Orange (KO)). **b.** FACS plots showing the cell cycle profiles at indicated time-points after the early S phase sort based on PI staining. n≥1000 cells measured per time-point from one experiment. **c.** Cumulative mitotic entry of unperturbed RPE Fucci cells sorted in early S phase and followed by time-lapse imaging after re-plating shows the highly synchronous progression through S/G2 phase. n=51 cells from one experiment. **d,e.** A

partial knock down of Emi1 was obtained by titration of siEmi1 in RPE *CCNB*^{YFP} cells (Final concentrations siRNA: 10, 5, 2.5, 1.25nM). Mitotic entry of undamaged G2 cells upon Luc- and Emi1-depletion is shown. Representative graph and western blot of two independent experiments. **f.** Line graphs of the data presented in figure 3d and e showing cumulative mitotic entry or cumulative Cyclin B1 degradation. Mean ± s.d. of three independent experiments. **g.** Line graphs of the data presented in figure 3f showing cumulative mitotic entry or cumulative Cyclin B1 degradation. Mean ± s.d. of three independent experiments. **h.** ^{Turq}EMI overexpression induced by doxycycline addition. Representative western blot of two independent experiments.

Figure S4

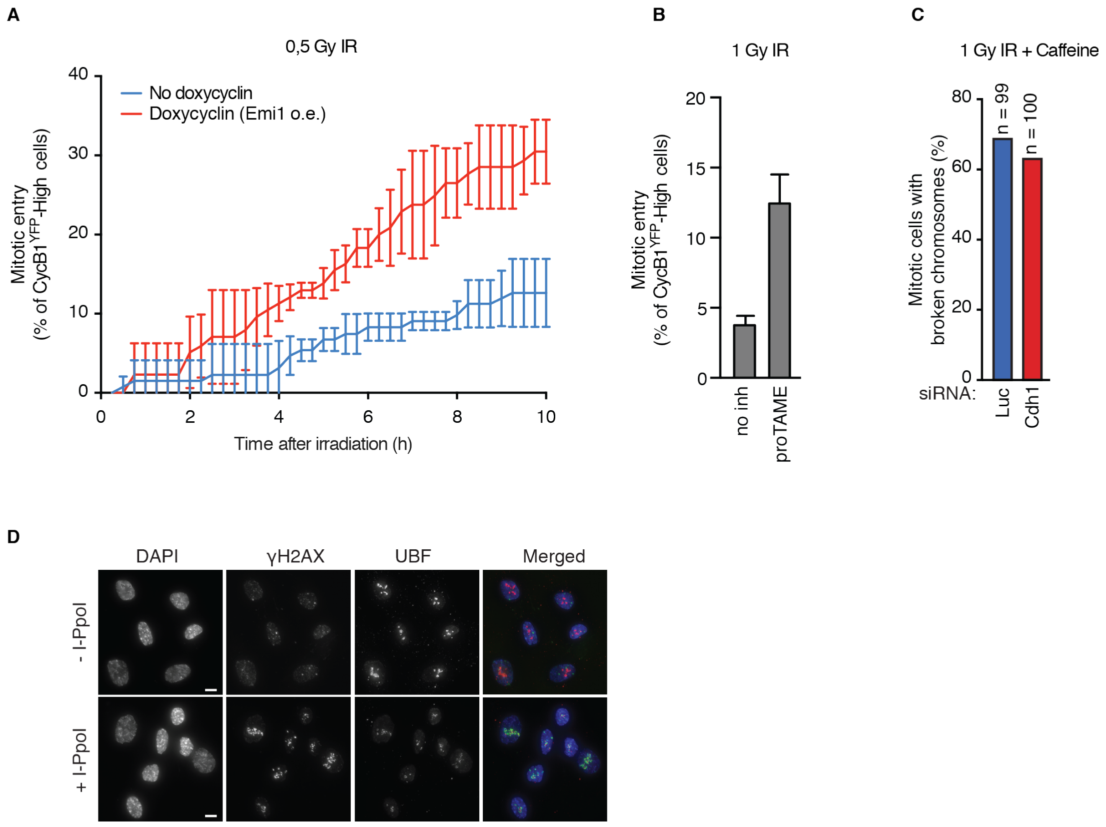

Supplementary figure 4: Hypersensitivity to DNA damage in antephase is needed to protect genomic stability a. Line graph of the data presented in figure 4d. Mean ± s.d. of three independent experiments. **b.** Mitotic entry of antephase cells (selected as in fig. 2a) within 15h following 1 Gy. The APC/C inhibitor proTAME was added just after IR. Mean ± s.e.m. of three independent experiments is shown. **c.** Quantification of mitotic cells with broken chromosomes as in figure 4g. Caffeine was added 30 min before IR to override DNA damage checkpoint signalling. Average of two independent experiments is shown. **d.** Stills showing nucleolar localized γH2AX, indicating rDNA damage upon I-PpoI induction. Scale bars, 10µm.

Figure S5

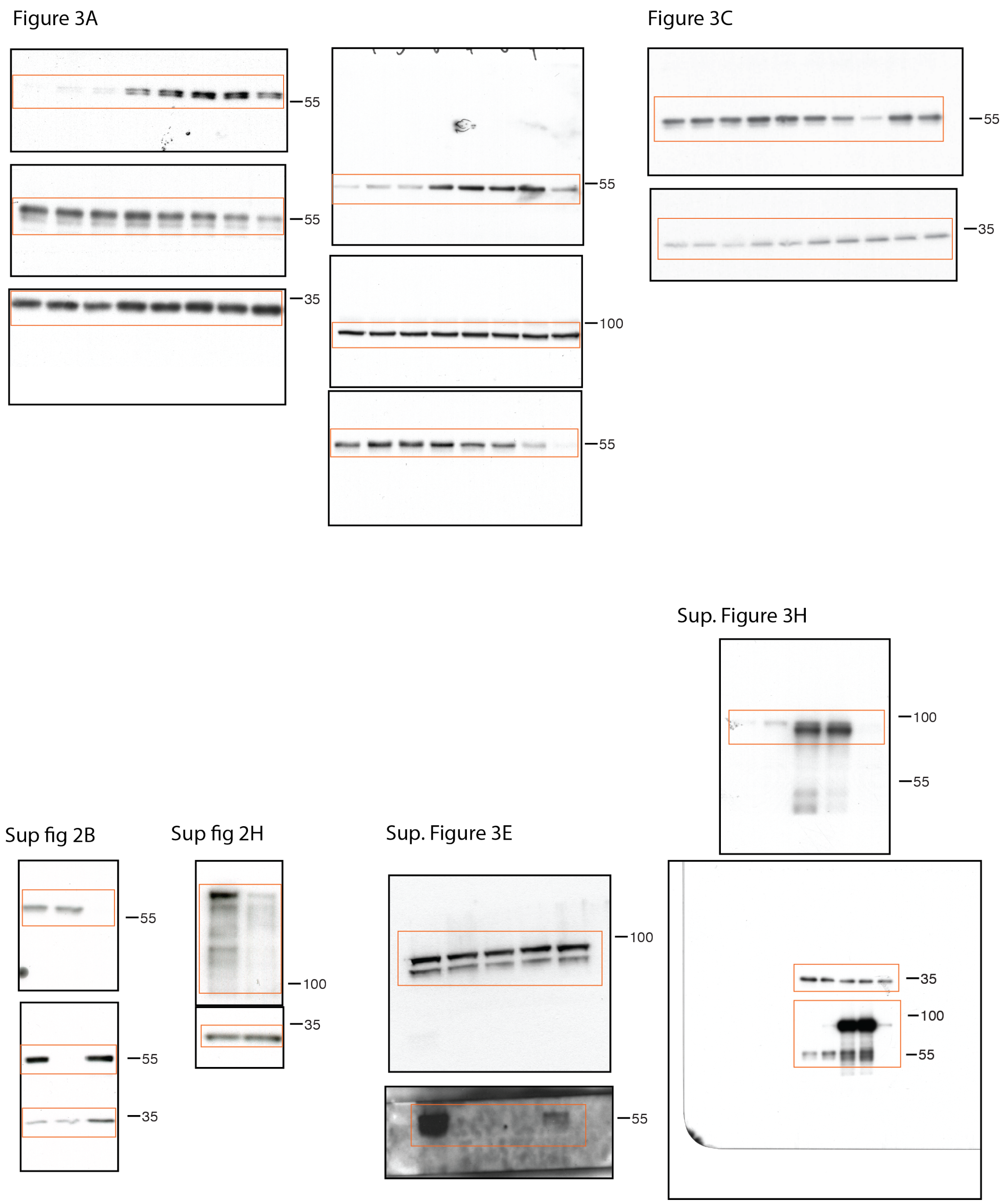

Supplementary figure 5:

Uncropped versions of western blots presented in this manuscript. Corresponding figures are indicated.

Supplementary methods

Spontaneous recovery analysis by FACS

Cells depleted for Emi1 using decreasing concentrations of siRNA (Final concentrations siRNA: 10, 5, 2.5, 1.25nM) were washed 24h after siRNA transfection with 1xPBS and cultured for 16h in medium containing BrdU (10µM) and nocodazole (250ng/ml). Cells were harvested and fixed in 70% ethanol (4˚C) for a minimum of 2 hours. In order to denature the DNA for BrdU staining, cells were incubated with 2M HCl for 15 minutes. Subsequently, the pH was neutralized with 0.1M Borate buffer (pH=8.5). Cells were stained with the primary antibodies: anti-BrdU and anti-pH3 (see methods) and corresponding secondary antibodies. Cells were treated with RNAse A (Sigma), and DNA was stained with Propidium Iodide. Cells were analysed using flow cytometry of 10^3 events (Cell Quest, Becton Dickinson).
