# Supplementary material for: Hypersensitivity to DNA damage in antephase as a safeguard for genome stability
Source: Nat Commun. 2016 Aug 26;7:12618. doi: 10.1038/ncomms12618 (PMC5007458; doi:10.1038/ncomms12618)
Supplement: Peer review file [file ncomms12618-s2.pdf]

Editorial Note: this manuscript has been previously reviewed at another journal that is not operating a transparent peer review scheme. This document only contains reviewer comments and rebuttal letters for versions considered at Nature Communications.
